# Barriers to Gene Flow in the Marine Environment: Insights from Two Common Intertidal Limpet Species of the Atlantic and Mediterranean

**DOI:** 10.1371/journal.pone.0050330

**Published:** 2012-12-11

**Authors:** Alexandra Sá-Pinto, Madalena S. Branco, Paulo B. Alexandrino, Michaël C. Fontaine, Stuart J. E. Baird

**Affiliations:** 1 Centro de Investigação em Biodiversidade e Recursos Genéticos (CIBIO/UP), Campus Agrário de Vairão, Vairão, Portugal; 2 Departamento de Zoologia-Antropologia, Faculdade de Ciências da Universidade do Porto, Porto, Portugal; 3 Ecologie, Systématique et Evolution, Université Paris-Sud, Orsay, France; 4 CNRS, Orsay, France; 5 Ecoanthropology and Ethnobiology UMR 5145 CNRS-MNHN-Université Paris 7 Musée de l'Homme, Paris, France; 6 Centre de Biologie et de Gestion des Populations (CBGP), Campus International de Baillarguet, CS 30 016, Montpelier/Lez, France; Biodiversity Insitute of Ontario - University of Guelph, Canada

## Abstract

Knowledge of the scale of dispersal and the mechanisms governing gene flow in marine environments remains fragmentary despite being essential for understanding evolution of marine biota and to design management plans. We use the limpets *Patella ulyssiponensis* and *Patella rustica* as models for identifying factors affecting gene flow in marine organisms across the North-East Atlantic and the Mediterranean Sea. A set of allozyme loci and a fragment of the mitochondrial gene cytochrome C oxidase subunit I were screened for genetic variation through starch gel electrophoresis and DNA sequencing, respectively. An approach combining clustering algorithms with clinal analyses was used to test for the existence of barriers to gene flow and estimate their geographic location and abruptness. Sharp breaks in the genetic composition of individuals were observed in the transitions between the Atlantic and the Mediterranean and across southern Italian shores. An additional break within the Atlantic cluster separates samples from the Alboran Sea and Atlantic African shores from those of the Iberian Atlantic shores. The geographic congruence of the genetic breaks detected in these two limpet species strongly supports the existence of transpecific barriers to gene flow in the Mediterranean Sea and Northeastern Atlantic. This leads to testable hypotheses regarding factors restricting gene flow across the study area.

## Introduction

Understanding the mechanisms that govern gene flow between locations is an essential issue for both scientists interested in evolution and speciation processes and ecological managers responsible for the implementation of sustainable management practices for exploited or endangered taxa. In the marine environment, high genetic homogeneity is expected across vast areas, as gene flow is assumed to occur over large geographic scales due to the lack of obvious barriers to dispersal and to the existence of pelagic larvae in many species [1], [2]. Furthermore the high population density of many marine species is expected to reduce drift, slowing genetic differentiation between populations, even in the total absence of gene flow [1]. Despite these expectations, sharp breaks in the genetic composition of individuals have been described for marine organisms across surprisingly small geographic distances, even for species with pelagic larvae. Various explanations have been put forward to explain these observations [2], such as: 1- high amounts of self recruitment [3], [4], [5], [6], [7], [8], 2- historical vicariance [9], [10] and 3- barriers to dispersal such as oceanic currents [11], [12], [13], [14], [15], 4- ecotones and differences in selective pressures [16], [13], [17] and 5- habitat discontinuities [11], [12], [18]. Such factors are likely to act together, promoting differentiation across a species' range [19], [13].

Here we studied patterns of gene flow in marine organisms across the North-East Atlantic (NEA) and Mediterranean Sea (MS). Regions of restricted gene flow have been reported in this area for many marine taxa [20], [21] but, except for the Atlantic-Mediterranean transition, these patterns are usually highly species dependent. Even across this strong biogeographic break, discordant patterns have been found among species with similar ecological requirements [22], [23], [24].

The limpets *Patella ulyssiponensis* Gmelin, 1791 and *Patella rustica* Linnaeus, 1758 were chosen as model organisms for the present study as these species *i*) have very limited mobility as adults, allowing patterns of genetic substructure to be more directly related to factors affecting larval dispersal; *ii*) they are very abundant and conspicuous on Mediterranean and NEA rocky shores. These two species have high census density and pelagic larvae which may spend up to 31.5 days in the water column [25], [26], [27] and thus might be expected to show little differentiation across their distribution range. The limited data available for the continental range of *P. ulyssiponensis* suggests no differentiation across Iberian Atlantic shores [28] and a pattern of isolation-by-distance (IBD) at the scale of NEA and MS [29]. For *P. rustica*, previous studies revealed high genetic homogeneity along the Iberian coast [28], [30] but strong genetic substructure within the MS, with two distinct forms meeting in South Italy where they form a hybrid zone [31].

In the present work we combined the use of clustering algorithms with geographic cline analyses to describe patterns of genetic variation over the range of these species and infer possible factors influencing gene flow. More specifically we aim to *i*) test if gene flow across this area is better explained by a large panmictic population, a pattern of IBD or if there is evidence for barriers to gene flow and *ii*) evaluate whether species show congruent geographic patterns. Our results show that gene flow across the study area is not homogenous suggesting the existence of barriers affecting multiple species.

## Methodology

### Sampling and Data collection

**Figure 1:**
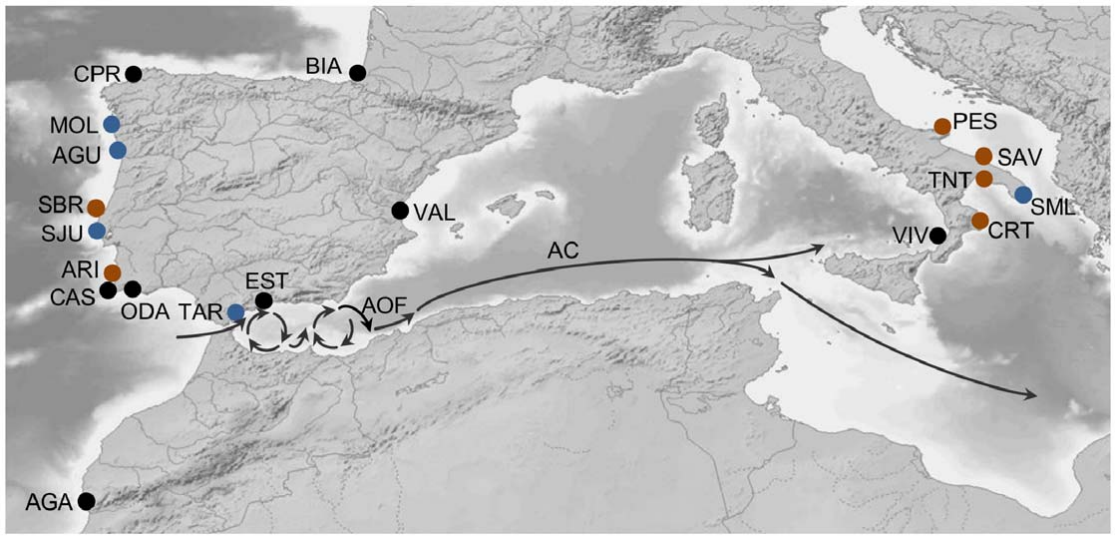
Study area and sampling sites. Arrows indicate the approximate location and direction of major sea surface currents. Blue dots: locations where only *Patella ulyssiponensis* individuals were sampled; brown dots: locations where only *Patella rustica* individuals were sampled; black dots: locations were both species were sampled. BIA: Biarritz; CPR: Cabo Prior; MOL: Moledo; AGU: Aguda; SBR: São Bernardino; SJU: São Julião; ARI: Arrifana; CAS: Castelejo; ODA: Olhos d'Água; AGA: Agadir; TAR: Tarifa; EST: Estepona; VAL: Valencia; VIV: Vibo Valentia; CRT: Crotone; TNT: Taranto; SML: Santa Maria di Leuca; SAV: Savelletri; PES: Peschici; AOF: Almeria-Oran front; AC: Algerian current.

The morphological taxonomy of limpets [32] is consistent with genetically distinct units [33], [28]. Samples of *P. rustica* and *P. ulyssiponensis*, identified according to this taxonomy were collected from 19 localities (Table 1, Figure 1), between 2002 and 2006. No specific permits were required for the described field studies. Individuals were transported in dry ice and preserved at −80°C. Data from samples used in previous studies were also included [34], [28], [35], [31].

**Table 1:** Sampled sites and number of individuals analysed for each data set.

Species	Localities	Sample size	
		allozymes	mtDNA
*Patella rustica* (Linnaeus, 1758)	Biarritz, France (BIA)	30^a^	4^b^+2^a^
	Cabo Prior, Spain (CPR)	30^a^	4^a^
	São Bernardino, Portugal (SBR)	30^a^	10^d^
	Arrifana, Portugal (ARI)	-	5^b^+2^a^
	Castelejo, Portugal (CAS)	33^c^	-
	Olhos de Água, Portugal (ODA)	30^a^	4^b^+2^a^
	Agadir, Morocco (AGA)	26^a^	14^d^
	Estepona, Spain (EST)	29^a^	11^d^
	Valencia, Spain (VAL)	30^e^	9^a^
	ViboValentia, Italy (VIV)	30^e^	9^a^
	Crotone, Italy (CRT)	30^e^	10^a^
	Taranto, Italy (TNT)	19^e^	6^a^
	Savelletri, Italy (SAV)	14^e^	5^a^
	Peschici, Italy (PES)	30^e^	5^a^
*Patella ulyssiponensis* (Gmelin, 1791)	Biarritz, France (BIA)	30^a^	8^a^
	Cabo Prior, Spain (CPR)	30^a^	5^a^
	Moledo, Portugal (MOL)	34^c^	-
	Aguda, Portugal (AGU)	30^c^	5^a^
	São Julião, Portugal (SJU)	-	5^b^+5^d^
	Castelejo, Portugal (CAS)	30^c^	2^b^+3^a^
	Olhos de Água, Portugal (ODA)	28^a^	5^a^
	Agadir, Morocco (AGA)	29^a^	11^d^
	Tarifa, Spain (TAR)	28^a^	3^b^+2^a^
	Estepona, Spain (EST)	30^a^	10^a^
	Valencia, Spain (VAL)	23^a^	11^d^
	ViboValentia, Italy (VIV)	25^a^	10^a^
	Santa Maria di Leuca, Italy (SML)	29^a^	7^a^

Data sources:

a present work; [34];

c [28];

d [35];

e [31].

For *P. rustica*, nine allozyme loci corresponding to eight enzymatic systems were analysed by starch gel electrophoresis: Malate dehydrogenase (MDH, EC : 1.1.1.37), Malic enzyme (ME, EC: 1.1.1.40), Isocitrate dehydrogenase (IDH, EC: 1.1.1.42), Glutamate-oxaloacetate transaminase (GOT, EC: 2.6.1.1), Phosphoglucomutase (PGM, EC: 2.7.5.1, two detectable loci hereafter referred to as PGM1 and PGM2), Peptidase D (PEP D, EC: 3.4.13.9), Glucosephosphateisomerase (GPI, EC: 5.3.1.9) and 6- Phosphogluconate dehydrogenase (PGD, EC: 1.1.1.44). For *P. ulyssiponensis* PGD was not scored and only one locus was detectable for PGM. Electrophoretic conditions, buffer systems and staining procedures are described elsewhere [28].

Total genomic DNA was extracted from a portion of foot muscle following standard procedures [36]. PCR amplification of mitochondrial gene cytochrome C oxidase subunit I was carried out according to Folmer *et al.* [37]. A fragment of approximately 670 base pairs (bp) was sequenced with the primers used in the PCR amplification.

### Data analyses

#### Allozyme data

Bayesian clustering algorithms which identify clusters of individual genotypes by maximising Hardy-Weinberg and linkage (HWL) equilibria ([38], [39], [40], [41] hereafter ‘clustering algorithms’) are powerful methodologies that allow an objective delimitation of even slightly differentiated populations. These may be used to identify barriers to gene flow and, in conjunction with clinal analyses, infer their possible causes. Clinal analyses are a long established way of studying gene flow [42], [43], [44], allowing us to test for the existence of barriers to gene exchange, infer their geographic location and properties, and to statistically compare such properties between taxa [45]. However, despite the extensive use of such methods in studies of hybrid zones, its application to the identification of ecological barriers to gene flow has been much more limited (but see [46]). In the present work, we combined the use of a clustering algorithm with likelihood analysis of geographic allele frequency clines [45] to test, identify and characterize barriers to gene flow. We chose this approach rather than alternatives [46], [47], [48], as the likelihood framework is particularly suited for testing hypothesis in comparative analyses for multiple species.

The clustering algorithm implemented in BAPS v4.14 [41], [49] was used to check for the existence of genetic clusters and to estimate the proportion of each individual's genome derived from each cluster. BAPS was chosen because it can co-estimate cluster membership and the number of clusters (K) during analysis and allows the clustering to be performed either at the individual level or at sampling location level. Pooling individuals within sampling locations increases statistical power in cases of low numbers of markers or weak genetic differentiation [50] but it assumes that distinct units are not mixed within each sampling location. To verify this assumption, HWL equilibria were tested within each locality sample using the exact-tests from GENEPOP v.3.1b [51]. Three analyses were performed with BAPS for each species: *i*) a spatial analysis at the individual level, *ii*) a spatial analysis at the level of sampling locations, *iii*) a non spatial analysis at the level of sampling locations. Runs were carried out for maximum K set to 5, 10 and 15. To check for consistency of admixture results, nine runs were performed for each species, with different numbers of iterations (100000, 5000, 2500) and different numbers of reference individuals (20, 30, 50). All analyses were performed with 20 instantiations of the selection of reference individuals.

Likelihood clinal analyses [52] were performed using information on admixture proportions inferred by BAPS: the average over individuals of the proportion of nuclear genome assigned to a cluster in each sampling location was used as a way of summarizing changes in genotype states over space. These proportions were plotted against the shortest distance by sea of each sampling location to Biarritz (in kilometres, km), the arbitrarily fixed origin for our marine coastal distance measures. In constructing the likelihood function for clinal analyses, observations (individual's cluster assignment proportions in *P. ulyssiponensis* and proportion of individual's genome assigned to the Western Mediterranean cluster in *P. rustica*) were assumed to be binomially distributed around the cline expectations for each locality (Likelihood = PDF [Binomial (*Ne*, *Pe*), *P_BAPs_* x *Ne*], where *Ne* is the effective sample size; *Pe* is the cline expectation; *P_BAPs_* is the estimated source frequency, cf. [53]). The BAPs estimates of source admixture proportions are calculated with neither credibility bounds nor support limits. We therefore make what we believe is a conservative assumption when estimating effective sample size: we regard the information about source over all the (semi diagnostic) loci combined as being one half the information available from a single diagnostic locus.

A model of clinal change was constructed that allows for two distinct sigmoid clines across the studied transect. The centres and widths of these two clines were independently estimated, with the proportion of genome assigned to a given cluster at the beginning of the second cline equal to that at the end of the first. Maximum likelihood estimates were calculated as in *Analyse* [54]. Support limit solutions and double cline hypotheses were treated in *Mathematica* v7.0 [55].

With the exception of Agadir (AGA), our sampling localities form a coastal-linear transect (Figure 1). Our cline models were developed for linear transects and, accordingly, Agadir's samples were excluded from clinal analyses. For each species, a hierarchy of nested hypotheses regarding the nature of allele frequency change was compared in order of increasing complexity, with more complex hypotheses being accepted only if their gain in likelihood was significant, given the increased number of parameters: *H_0_* - the data are explained by finite sampling from a panmictic system; *H_1_* - the data are explained by a single clinal change across the study area; *H_2_* - the data are explained by two distinct clinal changes across the studied area. Likelihood comparison is simplified by considering nested hypotheses: *H_0_* is nested within *H_1_* when the width of clinal change in *H_1_* becomes very wide with respect to the scale of the study region. Likewise *H_1_* is nested within *H_2_* when the second clinal change in *H_2_* becomes very wide with respect to the scale of the study region. To test if clines differed between species (regarding their centre location and width) we introduced another hypothesis: *H_3_* - the data are explained by two clines whose shape is shared (same centres and widths) between species. The likelihood of this hypothesis was then compared to the likelihood of the two species having two clines that differ in both their centre location and width. Likelihood ratio tests were used to compare alternative hypotheses.

The results obtained from the combined use of the clustering algorithm and clinal analyses were contrasted with *F*_st_-based comparisons of locality samples. ARLEQUIN 3.1 [56] was used to estimate *F*_st_ values between samples and to test their statistical significance (1000 permutations). The *F*_st_ values obtained between sampling locations from different groups were compared to those obtained between sampling locations from the same group using a randomisation test. For each test instance the Euclidean distance between the average *F*_st_ values obtained between and within given groups of sampling locations was calculated. Each sampling location was then randomly assigned to a group to obtain a randomised distribution of *F*_st_ distances between groups (1000 permutations). Significance was determined by comparing the observed *F*_st_ distance between groups with the randomised *F*_st_ distribution (cf [57]). This procedure was performed using POPTOOLS (www.cse.csiro.au/poptools/).

For both species, IBD was tested within each HWL cluster. IBD was not tested over the entire dataset, as it may confound the effect of cluster boundaries with the effect of distance [58]. The software IBD [59] was used to perform a Mantel test [60] comparing two matrices, the *F*_st_/(1−*F*_st_) and the log of the shortest distance by sea between each location, with 1000 replicates to test significance.

Genotypic data from allozyme loci for both *P. ulyssiponensis* and *P. rustica* is deposited in the Dryad repository: http://dx.doi.org/10.5061/dryad.8c26c.

#### mtDNA data

Sequences from the mtDNA gene cytochrome C oxidase subunit I were trimmed to a 600 bp fragment and aligned manually with Bioedit 7.0.9.0 [61]. All new haplotypes were submitted to EMBL Nucleotide Sequence Database (accession numbers HF547409 to HF547602). This data set was complemented with haplotypes already reported for the continental forms of these species, although, for some of them, no information was available regarding their frequency (Table 1). Parsimonious haplotype networks were estimated for *P. rustica* and *P. ulyssiponensis* with TCS (version 1.21, [62]). ARLEQUIN 3.1 [56] was used to calculate *p*-distance based *F*_st_ values between pairs of sampled localities and to test their statistical significance (1000 permutations). For both species, the pattern of IBD within population units was tested as previously described for allozymes. To test if *F*_st_ values obtained between sampling locations from different groups were significantly higher than those obtained between sampling locations from the same group, randomisation tests were performed as previously described.

## Results

### Allozymes

For *P. rustica* an average of 4.7±2.2 alleles were found per locus, with a maximum of eight alleles in PGM1 and a minimum of two for both PGD and MDH. For *P. ulyssiponensis* 5.9±2.3 alleles were found on average per locus, with a maximum of nine alleles at the GPI locus and a minimum of 3 at ME.

After applying Bonferroni's correction [63], no significant deviations from HWL equilibria were found for any species in any sampling location. Accordingly, the BAPS clustering algorithm was applied at both individual and locality levels. When BAPS was applied at the level of individuals, a single cluster was recovered for each species. In contrast, applied at the level of sampling locations, two clusters were recovered for *P. ulyssiponensis* and three for *P. rustica*, in both spatial and non-spatial analyses. The clustering differences between the individual and locality levels are consistent with the gain of information described by Corander *et al.* [50].

The three clusters recovered for *P. rustica* were the same for spatial (log marginal likelihood [LML]: −2447.8) and non-spatial analyses (LML: −2436.1). An “Atlantic” cluster (AtC) includes all the Atlantic sampling locations and Estepona; A “Western Mediterranean” cluster groups samples from Valência, Vibo Valentia, Crotone and Taranto; and an “Eastern Mediterranean” cluster unites samples from Savelletri and Peschici. The smallest change in the LML values when a sampling location is moved from the cluster to which it was assigned to another cluster was −10.9 for spatial analysis and −7.6 for non spatial analysis. These values are both of much higher magnitude than the significance limit of 2.3 proposed by Kass and Raftery [64] for Bayes Factors, thus lending strong support to the inferred groups. The three clusters showed significant differences in allelic frequencies at several allozyme markers with multilocus *F*_st_ values ranging from 0.08 to 0.20 (Table S1).

For *P. ulyssiponensis* two clusters were recovered in both spatial (LML: −2907,1) and non-spatial (LML: −2902.2) BAPS analyses. An “Atlantic” cluster (AtC) groups all the samples from the Atlantic together with Tarifa and Estepona. A “Mediterranean” cluster groups the remaining Mediterranean samples. The smallest change in the LML when a sampling location is moved from the cluster to which it was assigned to another cluster was −8.6 for spatial analysis and −7.5 for non-spatial analysis. Significant differences in allelic frequencies between these clusters were detected for ME, GPI and PEPD (Table S2).

Inference of admixture proportions was consistent over replicates so we report only those using 1×10^5^ iterations and 20 reference individuals.

**Figure 2:**
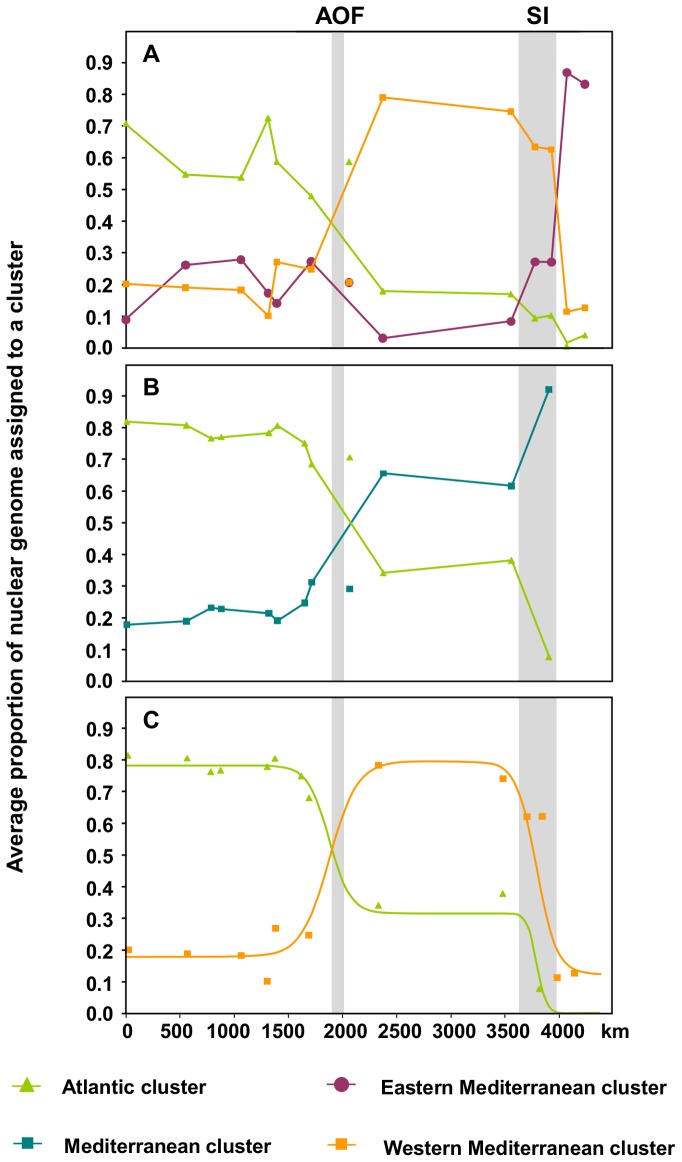
Geographic clines of BAPS clusters' proportions. Average proportion of nuclear genome in each sampling location assigned to one of the clusters recovered by BAPS for *Patella rustica* (**2A**) and *P. ulyssiponensis* (**2B**), plotted against their shortest sea distance to Biarritz. Points not connected by a line represent the sampling location of Agadir. **2C** - maximum likelihood sigmoid clines for the proportion of individual's genome assigned to the Atlantic cluster of *Patella ulyssiponensis* (green line) and to the Western Mediterranean cluster of *P. rustica* (orange line) obtained under the hypothesis of centres and widths being shared over the two species (H3); green and orange dots represent observations in *P. ulyssiponensis* and *P. rustica*, respectively. AOF – approximate location of Almeria-Oran Front; SI – South Italy.

Figure 2 shows the average proportion of individual's nuclear genotype assigned to a cluster in each sampling location, plotted against minimum marine distances to Biarritz. For *P. rustica* (Figure 2A) two steep changes in the genetic composition of the individuals can be observed. Although only two groups were recovered by the clustering analyses of *P. ulyssiponensis*, two steep breaks (delimiting three units) are also observed for this species (Figure 2B), roughly coincident with those reported for *P. rustica*. The break between Eastern and Western Mediterranean basins observed in *P. ulyssiponensis* is mostly driven by PEPD, the only locus showing significant *F*_st_ values between these two areas (see Table S3).

Clinal analyses showed data from both species was significantly better explained by the occurrence of two sigmoid clines across the sampling range rather than a single cline or sampling noise in a panmictic system (for *P. ulyssiponensis H_0_* is Rejected With Respect To (rwrt) H1 with ΔLL = 35.12, ΔDF = 3, p<0.001 and H1 is rwrt H2 with LL = 3.91, ΔDF = 3, p<0.05; for *P. rustica* H0 is rwrt H1 with ΔLL = 21.49, ΔDF = 3, p<0.001 and H1 is rwrt H2 with ΔLL = 23.55, ΔDF = 3, p<0.001). When H2 for each species is compared to the simplified hypothesis (H3) of cline shapes being shared between species, the complexity of H2 (species differences in cline shape) is not justified (ΔLL = 2.71, ΔDF = 4; p>0.24; but note that ΔLL would increase with less conservative estimates of effective sample size). Under the H3 hypothesis, the centre of the first cline coincides with the Almeria-Oran Front (1923 kms; 95%CI: 1733–2381 kms) while the second lies off South Italy (3896 kms; 95%CI: 3751–4003 kms; Figure 2).

**Figure 3.Genetic:**
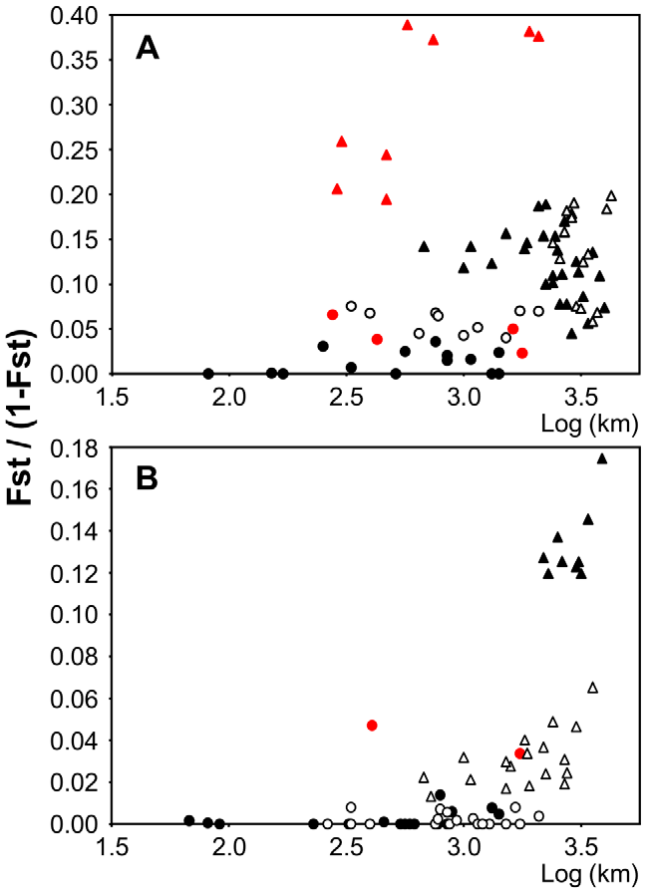
differentiation estimated from allozymes plotted against geographic distance. *F*_st_/(1−*F*_st_) obtained for the allozyme dataset of *Patella rustica* (**3A**) and *Patella ulyssiponensis*(**3B**) plotted against the log of the minimum marine distance between the sampling locations. Circles represent comparisons between locations within genetic clusters and triangles represent comparisons between locations from distinct clusters. For *P. rustica*: white circles - Agadir or Estepona versus the remaining Atlantic cluster (AtC) locations; red circles- Crotone or Taranto versus the remaining Western Mediterranean cluster (WMC) locations; white triangles - AtC locations versus Eastern Mediterranean cluster (EMC) locations, black triangles - AtC locations versus WMC locations and red triangles - WMC locations versus EMC locations. For *P. ulyssiponensis*: white circles - Agadir or Estepona versus the remaining AtC locations; red circles - Santa Maria di Leuca versus the remaining Mediterranean cluster (MC) locations; black triangles - Santa Maria di Leuca versus AtC locations; white triangles - AtC locations versus MC locations.

The *F*_st_ values obtained between sampling locations for *P. rustica* (Table 2) suggest strong genetic substructure across the study area except for samples from the Iberian Atlantic shores. According to our randomisation test, significantly higher *F*_st_ values were observed in comparisons involving samples from different genetic clusters, than in comparisons involving samples from the same cluster (*p*<0.01), independently of the distance between them (Figure 3A). For the samples assigned to the AtC, no significant correlation was found between genetic and geographic distances (*p* = 0.87). The *F*_st_ values between Agadir or Estepona and the remaining sampling locations from the AtC are significantly higher than those obtained for other intra-cluster comparisons (*p* = 0.04; Figure 3A).

**Table 2:** *F*_st_ values obtained between sampling locations of *Patella rustica* according to allozymes (lower diagonal) and mtDNA (upper diagonal).

	BIA	CPR	SBR	CAS	ARI	ODA	AGA	EST	VAL	VIV	CRT	TNT	SAV	PES
BIA	-	−0.21	-0.03	-	0.02	−0.10	0.05	0.10	0.00	−0.03	0.31*	0.85*	0.86*	0.90*
CPR	0.03*	-	−0.09	-	−0.07	−0.17	0.14	0.17*	0.02	−0.03	0.27	0.84*	0.86*	0.90*
SBR	0.02	−0.01	-	-	−0.03	−0.11	0.19*	0.22*	0.10	0.06	0.39*	0.86**	0.87**	0.90**
CAS	0.01	0.03*	0.03*	-	-	-	-	-	-	-	-	-	-	-
ARI	-	-	-	-	-	−0.04	0.35*	0.39*	0.25*	0.21*	0.39*	0.86**	0.88*	0.91*
ODA	−0.01	0.02	0.01	0.00	-	-	0.15	0.18*	0.05	0.01	0.32*	0.85*	0.86*	0.90*
AGA	0.07**	0.04*	0.04^**^	0.05**	-	0.06**	-	−0.01	−0.01	0.01	0.42*	0.89**	0.90**	0.93**
EST	0.06**	0.05**	0.04*	0.05**	-	0.07**	0.02	-	−0.01	0.01	0.38*	0.87**	0.89**	0.91**
VAL	0.09**	0.12**	0.10**	0.11**	-	0.10**	0.13**	0.11**	-	−0.06	0.33*	0.85**	0.86**	0.89**
VIV	0.12**	0.11**	0.11**	0.14**	-	0.13**	0.14**	0.12**	0.03*	-	0.34*	0.85**	0.86**	0.89**
CRT	0.10**	0.08**	0.07**	0.12**	-	0.10**	0.15**	0.15**	0.05*	0.06**	-	0.26	0.25	0.35*
TNT	0.07**	0.05*	0.04*	0.08**	-	0.07**	0.10**	0.08**	0.03	0.04*	0.01	-	−0.07	0.33*
SAV	0.16**	0.06*	0.07**	0.12**	-	0.14**	0.11**	0.13**	0.25**	0.26**	0.20**	0.14**	-	0.26
PES	0.16**	0.06**	0.07**	0.14**	-	0.15**	0.11**	0.12**	0.26**	0.26**	0.19**	0.16**	0.02	-

Location codes refer to Table 1; asterisks (*) indicate values significantly different from zero (p<0.05) and (**) indicate values that remain significant after Bonferroni correction [63].

The *F*_st_ values observed between sampling locations for *P. ulyssiponensis* were generally of lower magnitude than those observed for *P. rustica* (Table 3). The majority of significant *F*_st_ values were obtained for comparisons involving the Eastern Mediterranean location - Santa Maria di Leuca. Significantly higher *F*_st_ values were obtained between samples from different clusters, when compared to those obtained between samples from the same cluster (*p*<0.01), a pattern that cannot be explained by IBD (Figure 3B). This was true whether we considered two or three clusters (Santa Maria di Leuca considered as the third cluster). In fact, the comparisons involving Santa Maria di Leuca and the remaining sampling locations from the MC resulted in much higher *F*_st_ values than those obtained for the remaining intra-cluster comparisons (Figure 3B). Within the AtC, no significant correlation was found between genetic and geographic distances (p>0.84).

**Table 3:** *F*_st_ values obtained between sampling locations of *Patella ulyssiponensis* according to allozymes (lower diagonal) and mtDNA (upper diagonal).

	BIA	CPR	MOL	AGU	SJU	CAS	ODA	TAR	EST	AGA	VAL	VIV	SML
BIA	-	−0.09	-	0.13	0.17	−0.07	0.13	0.29*	0.56**	0.49**	0.11*	0.15*	0.13*
CPR	0.00	-	-	0.20	0.16	−0.03	0.17	0.30*	0.63**	0.51*	0.10*	0.14*	0.09*
MOL	0.03*	0.00	-	-	-	-	-	-	-	-	-	-	-
AGU	0.02	−0.00	0.00	-	−0.04	−0.00	−0.00	0.40	0.85*	0.60*	−0.06	−0.02	0.00
SJU	-	-	-	-	-	0.06	0.01	0.24*	0.63**	0.53**	0.02	0.07	0.08*
CAS	0.01	−0.01	−0.00	0.00	-	-	0.00	0.22	0.58*	0.47*	0.00	0.37	0.21
ODA	0.01	−0.01	−0.00	−0.00	-	0.00	-	0.17	0.63	0.41*	−0.04*	−0.00	0.01
TAR	0.01	0.00	0.00	0.01	-	−0.01	−0.00	-	0.26	0.15	0.17*	0.22*	0.16*
EST	0.00	−0.00	0.01	0.01	-	−0.01	0.01	0.01	-	−0.05	0.41**	0.48**	0.42**
AGA	0.01	−0.01	0.00	0.01	-	−0.01	0.01	−0.01	−0.00	-	0.37**	0.42**	0.37**
VAL	0.04	0.03	0.03*	0.03	-	0.02	0.03	0.02	0.02	0.02	-	0.01	0.02
VIV	0.06*	0.04	0.03	0.03	-	0.02	0.04	0.02	0.03*	0.02	−0.01	-	−0.03
SML	0.13**	0.11**	0.09**	0.10**	-	0.10**	0.11**	0.10**	0.10**	0.10**	0.03*	0.04*	-

Location codes refer to Table 1; asterisks (*) indicate values significantly different from zero (p<0.05) and (**) indicate values that remain significant after Bonferroni correction [63].

### mtDNA

**Figure 4:**
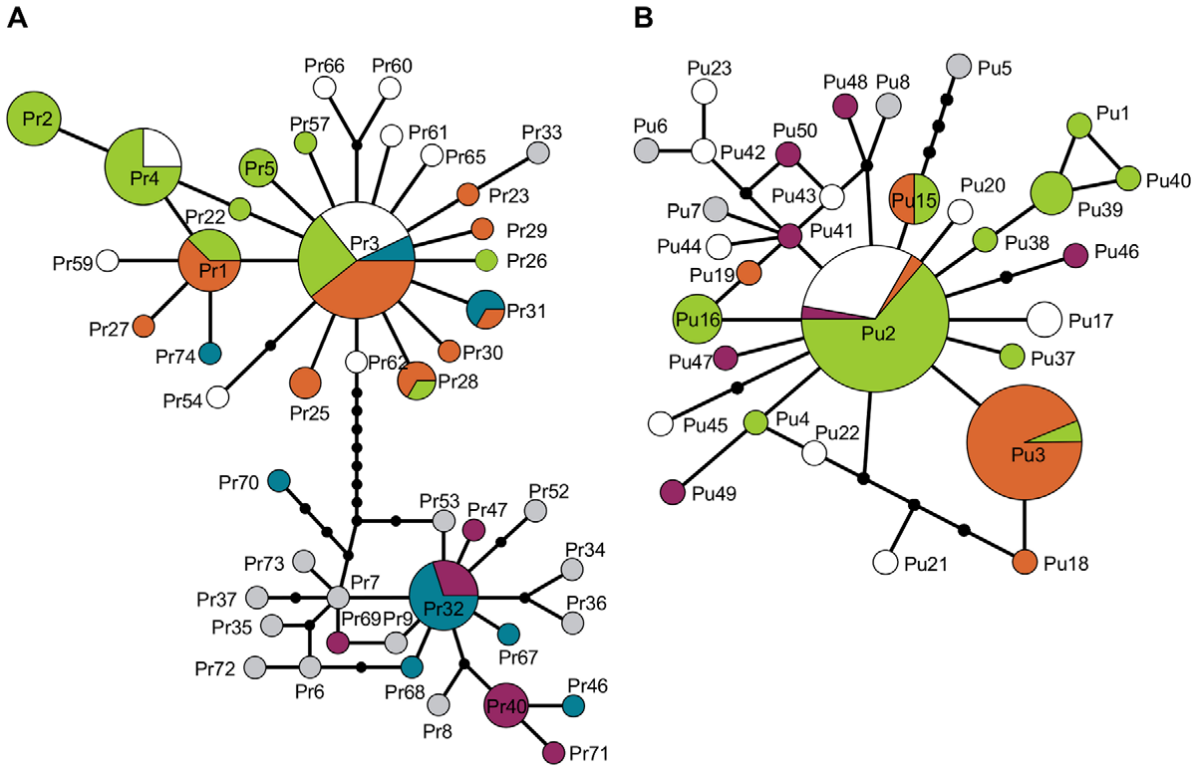
Haplotype networks of cytochrome c oxidase I of *Patella rustica* (4A) and *Patella ulyssiponensis* (4B). The area of each circle is proportional to haplotype frequency except for those represented by grey circles (haplotypes obtained from Genbank). For each haplotype, the area filled with a given colour is proportional to the number of individuals sampled in each of the given areas: Iberian Atlantic shores (green), Agadir or Estepona (orange), Western Mediterranean (white), Taranto or Crotone (blue), Eastern Mediterranean (purple). Black dots represent inferred missing haplotypes and full black lines connecting haplotypes or missing haplotypes represent a single mutation.

A total of 32 haplotypes were found for *P. rustica* samples included in the present work, of which 13 are first reported here. The TCS haplotype network is shown in Figure 4A (haplotype frequencies in Table S4). As previously reported, two geographically separate clades are present in the sampling area, meeting only in Crotone [34], [31]. High genetic differentiation between the two clades is reflected in the high *F*_st_ values obtained in comparisons between samples from the Eastern Mediterranean and the rest (Table 2). Intermediate *F*_st_ values were obtained in comparisons involving Crotone, the only sampling location where both clades are present. Within the AtC, there was no significant correlation between geographic distances and *F*_st_ values (p>0.36). The *F*_st_ values obtained in comparisons involving Agadir or Estepona and the remaining AtC sampling locations were significantly higher than those obtained for the comparisons within each of these two groups (p<0.05), a pattern not explained by IBD.

**Figure 5.Frequency:**
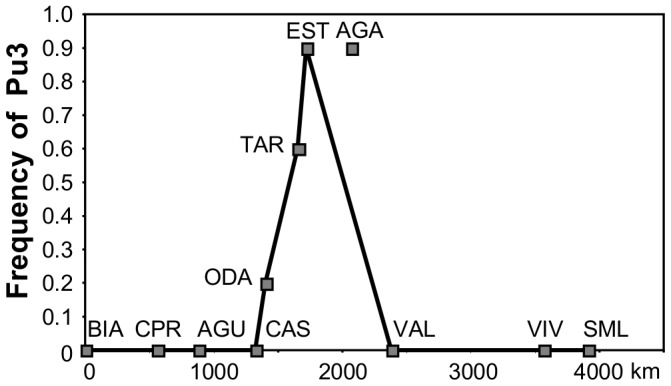
of the haplotype Pu3 of *Patella ulyssiponensis* plotted against the distance to Biarritz. The line represents the continuous coast and the point represents Agadir.

For *P. ulyssiponensis* 27 haplotypes were detected, 13 of which had already been reported [31], [34], [35]. The TCS haplotype network is shown in Figure 4B (haplotype frequencies in Table S5). Pu2 is the most common haplotype on western Iberian shores and on the northern shores of the Western Mediterranean but it is absent or at very low frequency in Agadir and Estepona where Pu3 reaches high frequencies (Figure 5). On the southern shores of the Iberian Peninsula Pu3 decreases in frequency from Estepona towards the west and it is also absent from Valencia, where Pu2 is again the most common haplotype. The changes in the frequencies of Pu2 and Pu3 are reflected in significantly higher *F*_st_ values for comparisons involving Agadir, Estepona and Tarifa with all other Atlantic samples (p<0.01; Table 3). Within the AtC, there was no significant correlation between geographic distances and *F*_st_ values (p>0.80).

## Discussion

Despite their pelagic larval stage and high population densities, *P. rustica* and *P. ulyssiponensis* show evidence of coincident barriers to gene flow within their ranges. Three lines of evidence favour the importance of these transpecific barriers in contrast to hypotheses of range-wide panmixis or IBD: *i*) clustering analyses detected more than one genetic cluster for both species; *ii*) significant sharp changes in the genetic composition of individuals were observed for both species across short geographic distances (Figure 2); *iii*) *F*_st_ comparisons of samples from different clusters were significantly higher than those obtained between samples from the same cluster (Figure 3). The sharp genetic discontinuities reported here can be interpreted as range boundaries delimiting evolutionary units. The genetic divergence between these evolutionary units may have been promoted by historical barriers that are currently absent from the study area, such as those caused by Pleistocene glaciations and associated sea level fluctuations (see review in [65]). On secondary contact of isolates a stable contact zone can be maintained by exogenous and/or endogenous selection [66]. However, even if maintenance is due wholly to endogenous selection, the location of the contact zone is expected to move toward, and be trapped at, extant barriers to gene exchange [67].The barriers here reported can include factors limiting dispersal as well as factors restricting survival and/or reproductive success of migrants. In this sense, sharp changes in environmental features may represent important barriers to gene exchange, as adaptation to distinct selective regimes can strongly reduce effective gene flow contributing to maintain sharp genetic clines at micro and macro-geographic scales ([66], [68] but see [69]). Distinguishing between barriers to dispersal and selection against migrants may however reveal a quite difficult task as in marine environment (and in our study area, see [65]) barriers to dispersal and strong ecological shifts tend to be associated in space [70].

### The Atlantic Mediterranean transition

The Atlantic-Mediterranean transition has long been recognised as a biogeographic break at both interspecific and intraspecific levels [71] with sharp genetic breaks reported for many other marine organisms [21], [24]. The transition also marks the range limits of the limpet species *Patella depressa* Pennant, 1777, *Patella ferruginea* Gmelin, 1791 and *Patella caerulea* Linnaeus, 1758 [32], [72]. Although these three species occur in the Gibraltar Strait, *P. depressa* is restricted to Atlantic shores while its closely related species *P. caerulea* [34], [35] as well as *P. ferruginea* are limited to Mediterranean shores [32], [72]. Our results show that gene flow across the Atlantic-Mediterranean transition is also restricted at intraspecific level, with congruent sharp breaks in allele frequencies occurring across this area for both *P. ulyssiponensis* and *P. rustica*. However, clinal analyses show the transition between the Atlantic and Mediterranean clusters is not located at the Gibraltar Strait (which lies outside the 95% confidence interval obtained for the cline centre) but most probably at the Almeria-Oran Front, with samples from Tarifa (1652 km from Biarritz, *P. ulyssiponensis*) and Estepona (1719 km from Biarritz, *P. rustica* and *P. ulyssiponensis*) being included in the Atlantic cluster. The Almeria-Oran Front (AOF, Figure 1) formed by the contact of Atlantic and Mediterranean waters (reviewed in [65]), has been proposed to restrict gene flow between Atlantic and Mediterranean populations of several marine species [22], [23], [21], [73], [24]. However, the Alboran Sea has attracted little sampling effort for intertidal organisms and only a few studies have clearly linked restricted gene flow between Mediterranean and Atlantic populations with the Almeria-Oran Front [74], [75], [76], [10], [73].

Several of the factors that restrict gene flow between populations of a given species are also expected to impose limits to species' ranges. These include: *i*) ecological shifts in the biotic and abiotic environment that restrict effective dispersal to unsuitable habitats or to a competitor's range (reviewed in [70], [77]) and *ii*) barriers to dispersal that prevent/slow down further dispersal [70] and where secondary contact zones tend to be trapped [67]. At the Atlantic-Mediterranean transition, patellid species boundaries seem not to be coincident with the intraspecific barriers to gene flow we report here suggesting that different factors are affecting species-internal versus -external boundaries. However, considering such small numbers of each category, it is quite likely the observed pattern could result from a random association of the two types of boundaries to two barrier factors. In this context, it would be interesting to test whether the AOF is also an effective intraspecific barrier to gene flow for *P. caerulea* and *P. ferruginea*, causing divergence between populations from the Alboran Sea and the remaining species range. Further research involving ecological modelling of species densities and larval dispersal would also contribute to an understanding of why the above mentioned species boundaries are close but not coincident with intraspecific barriers to gene flow.

### The Southern Italian barrier

Strong genetic substructure within the MS has been reported for various marine organisms, including fish [78], [79], [80], [81], [82], invertebrates [83], [84], [85], [31] and seagrasses [86], [87]. Despite sparse sampling within the Mediterranean region, our results also reveal genetic substructure. Significant sharp breaks in the genetic composition of individuals occur in both limpet species across southern Italian shores. Our results for *P. rustica* mirror those of Sá-Pinto *et al.* [31], who reported two highly divergent forms within this species that meet and hybridise along southern Italian shores. For *P. ulyssiponensis*, the clinal analyses revealed a significant increase in the average proportion of nuclear genome assigned to the Mediterranean cluster (Figure 2B and 2C) across the same geographic area, following change in the frequency of PEPD alleles. The coincidence of these changes in two species argues for the presence of a barrier limiting gene flow across this area. Further sampling in the Eastern Mediterranean may allow alternate hypotheses to be compared: an Eastern-Western Mediterranean differentiation maintained by oceanographic currents (as observed in seagrasses, [86], [87]) and/or distinct environmental conditions between the two basins; the existence of areas of unsuitable habitat (as suggested in [31]); or a reflection of more local patterns e.g. the differentiation of Adriatic populations [78], [79], [80], [82].

The transition between the Eastern and Western Mediterranean basins is also the limit of distribution for *P. ferruginea*, which is restricted to the Western Mediterranean [68], [88] and *Patella orientalis* Pallary 1938, restricted to the Eastern basin [31]. Thus the same factor(s) restricting gene flow in *P. rustica* and *P. ulyssiponensis* may also be affecting other limpet species, dictating their range limits. To test this hypothesis, it is necessary to study the distribution of these two species across this area with high geographic resolution and statistically compare their range boundaries with the data collected for *P. rustica* and *P. ulyssiponensis*. If the geographic clines (in gene frequency and species abundance) are congruent, this would argue for a common barrier promoting diversification and speciation in Mediterranean limpets by allowing population differentiation (as may be the case for *P. ulyssiponensis*) and/or restricting introgression between differentiated clades (as may be the case for *P. rustica*).

### Substructure within the Atlantic area

Within the AtC, no significant IBD was detected for either species. Our data suggest that there is, however, genetic differentiation, for both species, between two areas - one including Agadir and Estepona (and possibly Tarifa for *P. ulyssiponensis*) and the other including the remaining Atlantic sampling locations. In *P. ulyssiponensis* the distribution of the mtDNA haplotype Pu3 drives the high and significant *F*_st_ values obtained in comparisons of samples from the two areas. For *P. rustica*, comparisons also result in consistently higher *F*_st_ values for both allozymes and mtDNA.

To our knowledge this is the first time that this pattern of genetic substructure has been described for Atlantic intertidal organisms. The restricted gene flow between these two areas may well be explained by the lack of suitable habitat between them, as almost no rocky shores exist between Tarifa and Olhos d'Água. This hypothesis has already been proposed to explain genetic differentiation observed in *Stenosoma nadejda* Rezig, 1989 between Southern Portugal and the Gibraltar Strait [89]. The open ocean between Iberian and Atlantic African shores may reduce gene flow between these two areas. Sampling at finer geographic resolution of the Alboran Sea, the Gibraltar Strait and the Northwestern African shores, combined with dispersal and ecological modelling should allow this to be evaluated relative to alternative hypotheses.

### Concluding remarks

Our analytical methodology combining Bayesian clustering approaches and clinal analyses allowed us to identify transpecific barriers to gene flow across the NEA and MS. These barriers may be either promoting genetic divergence between populations or restricting introgression between differentiated forms [66], but in any case these represent boundaries separating units of evolutionary process of particular interest to evolutionary biologists and ecological managers. To test the role of historical processes in the observed genetic divergence, further studies are required that sample multiple nuclear and mtDNA sequences from each of the reported evolutionary units and estimate their divergence time, historical demography and migration rates. The present study also suggests clear sets of alternate hypotheses regarding causes of extant barriers. Future studies, combining finer geographic sampling resolution with simulations of larval dispersal and ecological modelling, would allow comparison of these barrier hypotheses. The identification of such factors is vital to both our understanding of marine biota evolution and the implementation of marine conservation strategies across NEA and MS.

## Supporting Information

Table S1 **Fst values obtained in comparisons between clusters obtained for** ***Patella rustica*** **.**
(DOC)

Table S2 **Fst values obtained in comparisons between clusters obtained for** ***Patella ulyssiponensis*** **.**
(DOC)

Table S3 **Fst values obtained in comparisons between the three geographic areas delimited by the barriers identified by clinal analyses for** ***Patella ulyssiponensis*** **.**
(DOC)

Table S4 **mtDNA haplotype frequencies in each sampling location for** ***Patella rustica*** **.**
(DOC)

Table S5 **mtDNA haplotype frequencies in each sampling location for** ***Patella ulyssiponensis*** **.**
(DOC)
