# Supplementary material for: Barriers to Gene Flow in the Marine Environment: Insights from Two Common Intertidal Limpet Species of the Atlantic and Mediterranean
Source: PLoS One. 2012 Dec 11;7(12):e50330. doi: 10.1371/journal.pone.0050330 (PMC3519802; doi:10.1371/journal.pone.0050330)
Supplement: Table S1 — Fst values obtained in comparisons between clusters obtained for Patella rustica . (DOC) [file pone.0050330.s001.doc]

Table S1. Fst values obtained in comparisons between clusters obtained for *Patella rustica*.

| Species | Allozyme | Region | | |
| --- | --- | --- | --- | --- |
|  |  |  | Atlantic | Med West |
| *Patella rustica* | PEPD | Med West | 0.036** | - |
|  |  | Med East | 0.091** | 0.020* |
|  | GOT | Med West | 0 | - |
|  |  | Med East | 0 | 0 |
|  | GPI | Med West | 0.100** | - |
|  |  | Med East | 0.110** | 0.002 |
|  | IDH | Med West | 0 | - |
|  |  | Med East | 0 | 0 |
|  | MDH | Med West | 0.002 | - |
|  |  | Med East | 0.013 | 0.007 |
|  | ME | Med West | 0.007 | - |
|  |  | Med East | 0.003 | 0.020* |
|  | PGD | Med West | 0.000 | - |
|  |  | Med East | 0.008 | 0.005 |
|  | PGM1 | Med West | 0.020* | - |
|  |  | Med East | 0.003 | 0.008 |
|  | PGM2 | Med West | 0.130** | - |
|  |  | Med East | 0.130** | 0.360** |
|  | TOTAL | Med West | 0.077** | - |
|  |  | Med East | 0.083** | 0.201** |
|  |  |  |  |  |
|  |  |  |  |  |

An asterisk (*) indicate values significantly different from zero (p<0.05) and (**) indicate values that remain significant after Bonferroni correction (Rice, 1989).
