# Supplementary material for: Barriers to Gene Flow in the Marine Environment: Insights from Two Common Intertidal Limpet Species of the Atlantic and Mediterranean
Source: PLoS One. 2012 Dec 11;7(12):e50330. doi: 10.1371/journal.pone.0050330 (PMC3519802; doi:10.1371/journal.pone.0050330)
Supplement: Table S2 — Fst values obtained in comparisons between clusters obtained for Patella ulyssiponensis . (DOC) [file pone.0050330.s002.doc]

Table S2. Fst values obtained in comparisons between clusters obtained for *Patella ulyssiponensis*.

| Species | Allozyme | Region | | |
| --- | --- | --- | --- | --- |
|  |  |  |  |  |
|  |  |  | Mediterranean | |
|  | PEPD | Atlantic | 0.048** | |
|  | PGM | Atlantic | 0.012* | |
|  | GOT | Atlantic | 0 | |
|  | GPI | Atlantic | 0.056** | |
|  | IDH | Atlantic | 0.008 | |
|  | MDH | Atlantic | 0.012 | |
|  | ME | Atlantic | 0.101** | |
|  | TOTAL | Atlantic | 0.048** | |

An asterisk (*) indicate values significantly different from zero (p<0.05) and (**) indicate values that remain significant after Bonferroni correction (Rice, 1989).
