# Supplementary material for: Barriers to Gene Flow in the Marine Environment: Insights from Two Common Intertidal Limpet Species of the Atlantic and Mediterranean
Source: PLoS One. 2012 Dec 11;7(12):e50330. doi: 10.1371/journal.pone.0050330 (PMC3519802; doi:10.1371/journal.pone.0050330)
Supplement: Table S3 — Fst values obtained in comparisons between the three geographic areas delimited by the barriers identified by clinal analyses for Patella ulyssiponensis . (DOC) [file pone.0050330.s003.doc]

Table S3. Fst values obtained in comparisons between the three geographic areas delimited by the barriers identified by clinal analyses for *Patella ulyssiponensis*.

| Species | Allozyme | Region | | |
| --- | --- | --- | --- | --- |
|  |  |  | Atlantic | Med West |
| *Patella ulyssiponensis* | PEPD | Med West | 0.006 | - |
|  |  | Med East | 0.176** | 0.124** |
|  | PGM | Med West | 0.006 | - |
|  |  | Med East | 0.015 | 0 |
|  | GOT | Med West | 0 | - |
|  |  | Med East | 0.010 | 0.009 |
|  | GPI | Med West | 0.050** | - |
|  |  | Med East | 0.050** | 0 |
|  | IDH | Med West | 0.005 | - |
|  |  | Med East | 0.001 | 0.000 |
|  | MDH | Med West | 0.011* | - |
|  |  | Med East | 0.007 | 0.001 |
|  | ME | Med West | 0.070** | - |
|  |  | Med East | 0.144** | 0.013 |
|  | TOTAL | Med West | 0.028** | - |
|  |  | Med East | 0.090** | 0.036** |

An asterisk (*) indicate values significantly different from zero (p<0.05) and (**) indicate values that remain significant after Bonferroni correction (Rice, 1989).
